# The Genome Sequence of a Type ST239 Methicillin-Resistant *Staphylococcus aureus* Isolate from a Malaysian Hospital

**DOI:** 10.4056/sigs.3887716

**Published:** 2014-04-20

**Authors:** LS Lee, LK Teh, ZF Zainuddin, MZ Salleh

**Affiliations:** 1 Integrative Pharmacogenomics Centre, Faculty of Pharmacy, Universiti Teknologi MARA Malaysia, 42300 Bandar Puncak Alam, Selangor, Malaysia.; 2 School of Health Sciences, Universiti Sains Malaysia, 16150 Kubang Kerian, Kelantan, Malaysia; 3 School of Health Sciences, Universiti Sains Malaysia, 16150 Kubang Kerian, Kelantan, Malaysia

**Keywords:** *Staphylococcus aureus*, MRSA, Malaysia, Genomics

## Abstract

We report the genome sequence of a healthcare-associated MRSA type ST239 clone isolated from a patient with septicemia in Malaysia. This clone typifies the characteristics of ST239 lineage, including resistance to multiple antibiotics and antiseptics.

## Introduction

**Figure 1:**
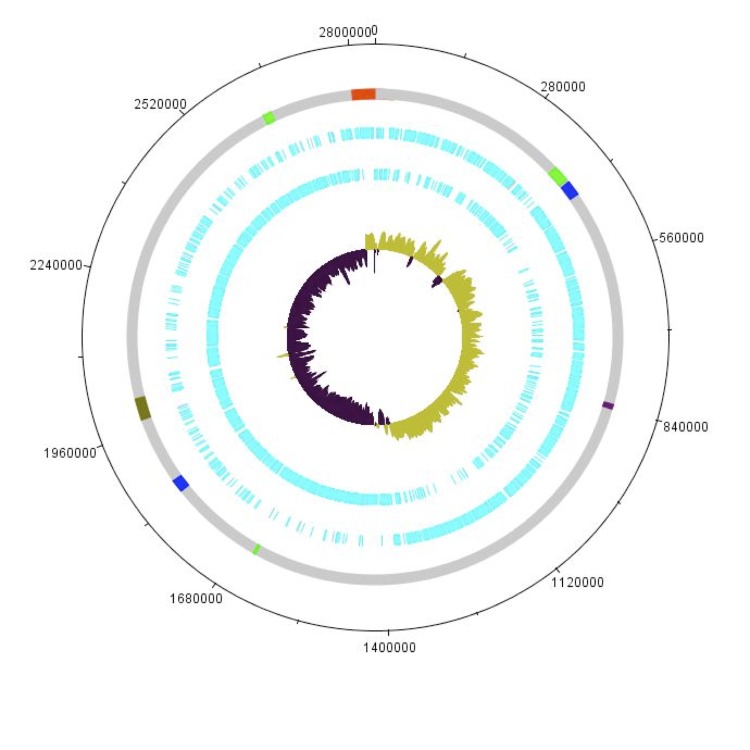
Phylogenetic tree highlighting the position of *Staphylococcus aureus* strain PR01 relative to other type strains within the *Staphylococcaceae*. The strains and their corresponding GenBank accession numbers for 16S rRNA genes are: *S. aureus* strain ATCC 12600, L36472; *S. saprophyticus* strain ATCC 15305, AP008934; *S. epidermidis* strain ATCC 14990, D83363; *S. hominis* strain DSM 20328, X66101; *S. haemolyticus* strain CCM2737, X66100; and *S. cohnii* strain ATCC 49330, AB009936. The tree uses sequences aligned by the RDP aligner, and uses the Jukes-Cantor corrected distance model to construct a distance matrix based on alignment model positions without the use of alignment inserts, and uses a minimum comparable position of 200. The tree is built with RDP Tree Builder, which uses Weighbor [1] with an alphabet size of 4 and length size of 1000. The building of the tree also involves a bootstrapping process repeated 100 times to generate a majority consensus tree [2]. *Staphylococcus lutrae* (X84731) was used as an outgroup.

Antibiotic resistance in *S. aureus* is a major concern, as an increasing number of infections are caused by methicillin-resistant *S. aureus* (MRSA). Figure 1 shows the phylogenetic position of *S. aureus* in relation to other staphylococci. In Malaysia, the incidence of MRSA-related infections is a cause of concern in hospitals country-wide. Health-associated MRSA (HA-MRSA) has been dominated by a few lineages in Southeast Asia, particularly ST239. Sequence type 239 is an international healthcare-associated (HA) MRSA lineage prevalent in Asia, South America and Eastern Europe, which includes EMRSA-1, -4, -7, and -11 and the Brazilian, Portuguese, Hungarian, and Viennese clones. Strains of type ST239 are typically resistant to multiple classes of antibiotics and antiseptics such as β-lactam antibiotics.

## Classification and features

We have chosen a representative of an MRSA strain, termed MRSA PR01 isolated from a patient with septicemia, isolated from a hospital in Kuala Lumpur. Table 1 indicates general information gathered on MRSA PR01. The MRSA PR01 strain has been identified as sequence type 239 (ST239) by multilocus sequence typing (MLST). Initial disc susceptibility tests showed that the strain is resistant to β-lactam antibiotics oxacillin, ampicillin, cefuroxime, ceftriaxone, gentamicin, erythromycin, ciprofloxacin and co-trimoxazole.

**Table 1:** Classification and general features of *Staphylococcus aureus* MRSA PR01

**MIGS ID**	**Property**	**Term**	**Evidence code**^a^
	Current classification	Domain *Bacteria* Phylum *Firmicutes* Class *Bacilli* Order *Bacillales* Family *Staphylococcaceae* Genus *Staphylococcus* Species *Staphylococcus aureus* Type strain MRSA PR01	[3] [4-7] [8,9] [6,10] [9,11] [6,12] [6,12] TAS
	Gram stain	Positive	TAS
	Cell shape	Coccus	TAS
	Motility	Non-motile	TAS
	Sporulation	Non-sporulating	TAS
	Temperature range	Mesophile	TAS
	Optimum temperature	30-37°C	TAS
	Carbon source	Glucose	TAS
	Energy source	Chemoorganotrophic	
	Terminal electron receptor		
MIGS-6	Habitat	Human respiratory tract, skin	TAS
MIGS-6.3	Salinity		
MIGS-22	Oxygen	Facultative anaerobe	TAS
MIGS-15	Biotic relationship		
MIGS-14	Pathogenicity	Opportunistic pathogen	TAS
MIGS-4	Geographic location	Malaysia	IDA
MIGS-5	Sample collection time	May 2009	IDA
MIGS-4.1	Latitude	4.1936°N	IDA
MIGS-4.2	Longitude	103.7249°E	IDA
MIGS-4.3	Depth	Not reported	IDA
MIGS-4.4	Altitude	Not reported	IDA

a) Evidence codes - IDA: Inferred from Direct Assay; TAS: Traceable Author Statement (i.e., a direct report exists in the literature); NAS: Non-traceable Author Statement (i.e., not directly observed for the living, isolated sample, but based on a generally accepted property for the species, or anecdotal evidence). These evidence codes are from the Gene Ontology project [13].

## Genome sequencing information

### Genome project history

This organism was selected for sequencing as a representative of MRSA infection in a local Malaysian hospital. The genome sequences of this organism were deposited in GenBank (WGS database). Sequencing, finishing and annotation were performed at the Integrative Pharmacogenomics Centre (PROMISE), UiTM. Table 2 presents the project information and its association with MIGS version 2.0 compliance [14].

**Table 2:** Project information

**MIGS ID**	**Property**	**Term**
MIGS-31	Finishing quality	Non-contiguous Finished
MIGS-28	Libraries used	One 350bp Illumina GAIIx genomic library
MIGS-29	Sequencing platforms	Illumina GAIIx, Sanger
MIGS-31.2	Fold coverage	>200×
MIGS-30	Assemblers	CLCBio Genomics Workbench
MIGS-32	Gene calling method	Glimmer and GeneMark
	Genome Database release	DDBJ/EMBL/Genbank/
	Genbank ID	ANPO01000000
	Genbank Date of Release	January 11, 2014
	GOLD ID	
	Project relevance	Medical, Tree of life

### Growth conditions and DNA isolation

MRSA PR01 was grown overnight under aerobic conditions in Tryptic Soy Broth at 37°C. DNA extraction was performed using MasterPure™ Gram Positive DNA Purification Kit (Epicentre, Madison, USA) as per manufacturer's instructions. The concentration and purity of resultant DNA was assessed by UV spectrophotometry (Nanodrop, Thermo Scientific). 5 µg of genomic DNA (A_260/280_ = 1.88) was used for library preparation.

### Genome sequencing and assembly

The genome sequence was obtained using 104 Mb of paired-end (300 bp spacing) data from the Illumina GA*II_x_* platform (Illumina, San Diego, CA) with 36-bp reads. Sequence data were assembled using CLCBio Genomics Workbench (CLC bio, Aarhus, Denmark). One hundred and ninety-five contigs (N50: 13,272 bp) were generated, and were overlaid with the reference sequence Mu50 using OSLay. Fourteen supercontigs were generated as a result. Gaps were closed using Sanger sequencing.

### Genome annotation

The genome was annotated using BASys [15] and RAST [16].

## Genome properties

**Figure 2:**
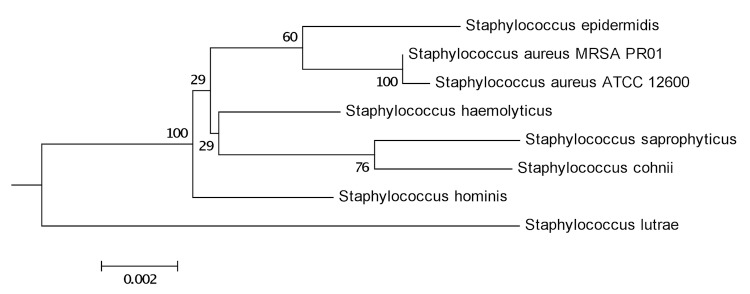
Visual representation of the MRSA PR01 genome. From outer to inner tracks: Scale (in bases); annotated CDSs colored according to predicted function (refer to legend); forward strand CDS; reverse strand CDS; GC skew.

The MRSA PR01 genome consists of a 2,725,110-bp circular chromosome with a GC content of 32.6% (Table 3). The MRSA PR01 genome contains 2668 CDs with 19 rRNA features (). A total of 1722 (64.5%) of protein coding genes were assigned to COGs, and a breakdown of the functional assignment of COG-assigned genes is shown in Table 4. Plasmid sequences were only partially sequenced. Figure 2 depicts genomic regions of interest found in the preliminary analysis of the MRSA PR01 genome.

**Table 3:** Nucleotide content and gene count levels of the MRSA PR01 genome

Attribute	Value	% of total^a^
Genome size (bp)	2,725,110	
DNA G+C content (bp)	888,386	32.6
Total genes	2687	
RNA genes	19	0.7
Protein-coding genes	2668	99.3
Genes assigned to COGs	1722	64.5

a) The total is based on either the size of the genome in base pairs or the total number of protein coding genes in the annotated genome.

**Table 4:** Number of genes associated with the 25 general COG functional categories

**Code**	**Value**	**%age**^a^	**Description**
J	140	5.247	Translation
A	-	-	RNA processing and modification
K	127	4.760	Transcription
L	126	4.723	Replication, recombination and repair
B	-	-	Chromatin structure and dynamics
D	23	0.862	Cell cycle control, mitosis and meiosis
Y	-	-	Nuclear structure
V	-	-	Defense mechanisms
T	47	1.762	Signal transduction mechanisms
M	91	3.411	Cell wall/membrane biogenesis
N	4	0.150	Cell motility
Z	0	0	Cytoskeleton
W	0	0	Extracellular structures
U	0	0	Intracellular trafficking and secretion
O	72	2.699	Posttranslational modification, protein turnover, chaperones
C	106	3.973	Energy production and conversion
G	129	4.835	Carbohydrate transport and metabolism
E	186	6.972	Amino acid transport and metabolism
F	68	2.549	Nucleotide transport and metabolism
H	83	3.111	Coenzyme transport and metabolism
I	62	2.324	Lipid transport and metabolism
P	123	4.610	Inorganic ion transport and metabolism
Q	23	0.862	Secondary metabolites biosynthesis, transport and catabolism
R	193	7.234	General function prediction only
S	119	4.460	Function unknown
-	946	35.457	Not in COGs

a) The total is based on the total number of protein coding genes in the annotated genome.

Initial analysis of the genome revealed several key features. This genome has a typical SCCmec type III cassette, containing cadmium resistance genes. SCCmec type III is a composite element that is comprised of SCCmec and SCCmercury. In the MRSA PR01 genome, like others, this region harbors *ccrC*, pI258 and Tn554 as well as the genes involved in cadmium resistance. The MRSA PR01 genome contains two pathogenicity islands, and several resistance features were identified such as the *qacA* gene, which confers resistance to antiseptics such as cationic biocides, quaternary ammonium salts, and diamidines via an export-mediated mechanism, and the *norA* gene which confers resistance to hydrophilic quinolones such as norfloxacin and ciprofloxacin. There were 9 regions defined as prophage regions by PHAST [17] with one complete prophage region.

## Conclusion

This study is the first to report on the whole genome sequence of a Malaysian MRSA isolate. Preliminary analysis of the genome has highlighted the genetic determinants that are responsible for the organism to adapt easily to selective pressures. Further research is being conducted to provide insight on the adaptive power of this healthcare-associated strain to attain high resistance to antibiotics.

*Nucleotide sequence accession numbers*. This Whole Genome Shotgun project has been deposited at DDBJ/EMBL/GenBank under the accession ANPO00000000. The version described in this paper is the first version, ANPO01000000.
